# Age-Related Alterations in Electroencephalography Connectivity and Network Topology During n-Back Working Memory Task

**DOI:** 10.3389/fnhum.2018.00484

**Published:** 2018-12-06

**Authors:** Fengzhen Hou, Cong Liu, Zhinan Yu, Xiaodong Xu, Junying Zhang, Chung-Kang Peng, Chunyong Wu, Albert Yang

**Affiliations:** ^1^Key Laboratory of Biomedical Functional Materials, School of Science, China Pharmaceutical University, Nanjing, China; ^2^School of Foreign Languages and Cultures, Nanjing Normal University, Nanjing, China; ^3^Department of TCMs Pharmaceuticals, School of Traditional Chinese Pharmacy, China Pharmaceutical University, Nanjing, China; ^4^Division of Interdisciplinary Medicine and Biotechnology, Department of Medicine, Beth Israel Deaconess Medical Center, Harvard Medical School, Boston, MA, United States; ^5^Key Laboratory of Drug Quality Control and Pharmacovigilance, Ministry of Education, China Pharmaceutical University, Nanjing, China; ^6^Department of Pharmaceutical Analysis, China Pharmaceutical University, Nanjing, China

**Keywords:** aging, graph theory, working memory, phase lag index, electroencephalography

## Abstract

The study of the healthy brain in elders, especially age-associated alterations in cognition, is important to understand the deficits created by Alzheimer's disease (AD), which imposes a tremendous burden on individuals, families, and society. Although, the changes in synaptic connectivity and reorganization of brain networks that accompany aging are gradually becoming understood, little is known about how normal aging affects brain inter-regional synchronization and functional networks when items are held in working memory (WM). According to the classic Sternberg WM paradigm, we recorded multichannel electroencephalography (EEG) from healthy adults (young and senior) in three different conditions, i.e., the resting state, 0-back (control) task, and 2-back task. The phase lag index (PLI) between EEG channels was computed and then weighted and undirected network was constructed based on the PLI matrix. The effects of aging on network topology were examined using a brain connectivity toolbox. The results showed that age-related alteration was more prominent when the 2-back task was engaged, especially in the theta band. For the younger adults, the WM task evoked a significant increase in the clustering coefficient of the beta-band functional connectivity network, which was absent in the older adults. Furthermore, significant correlations were observed between the behavioral performance of WM and EEG metrics in the theta and gamma bands, suggesting the potential use of those measures as biomarkers for the evaluation of cognitive training, for instance. Taken together, our findings shed further light on the underlying mechanism of WM in physiological aging and suggest that different EEG frequencies appear to have distinct functional correlates in cognitive aging. Analysis of inter-regional synchronization and topological characteristics based on graph theory is thus an appropriate way to explore natural age-related changes in the human brain.

## Introduction

Memory decline is typically the first symptom noticed in patients with Alzheimer's disease (AD). More than 35 million people worldwide have been diagnosed with AD, making it the most common form of dementia and a tremendous burden on individuals, families, and society (Querfurth and Laferla, 2010). The principal risk factor for AD is age, with incidence doubling every 5 years after the age of 65 years (Querfurth and Laferla, 2010). The study of the healthy brain aging, especially age-related changes in memory, is vitally important to understand the deficits created by AD.

Working memory (WM) involves the ability to maintain and manipulate information over short periods of time. It can be subdivided into two parts: the initial encoding of information, and maintenance and retrieval of WM items (Roux and Uhlhaas, 2014). Much larger age differences are seen for WM tasks than for short-term memory tasks that require only storage and maintenance of information (Bopp and Verhaeghen, 2009). One of the most popular experimental paradigms for WM studies has been the n-back task. In this task, participants are asked to monitor the identity or location of a series of verbal or non-verbal stimuli, and to indicate whether the currently presented stimulus is the same as the one presented previously (Owen et al., 2005). Much effort has been made to elucidate the age-related alterations of brain characteristics during n-back tasks and to uncover their underlying mechanisms using functional neuroimaging techniques such as functional magnetic resonance imaging (fMRI) (Lamar et al., 2004; Mattay et al., 2006; Schulze et al., 2011; Heinzel et al., 2014; Li et al., 2015; Dev et al., 2017; Jacobs et al., 2017) and electroencephalography (EEG) (Missonnier et al., 2004, 2011; Pesonen et al., 2007; Ho et al., 2012; Saliasi et al., 2013; Barr et al., 2014; Gajewski and Falkenstein, 2014; Dong et al., 2015; Padgaonkar et al., 2017).

Among these techniques, EEG has the advantages of easy accessibility and excellent temporal resolution. It is also a more sensitive approach for rapid cognitive processes such as those involved in WM, in which changes occur on a time scale of several 100 ms. Analysis of EEG results allows researchers to obtain information on brain functioning during different behavioral and cognitive states. In addition, neural oscillations at specific frequencies have been shown to be related to certain cognitive processes (Roux and Uhlhaas, 2014). For example, there is considerable evidence that theta-band activity is associated with the processes involved in memory, and increased theta activity in frontal areas is a common EEG alteration in studies of WM (Jensen and Tesche, 2010). However, the functional role of distinct EEG oscillations, typically involving theta, alpha, beta, and gamma activity, and their relationship to WM processes in aging has remained unclear (Roux and Uhlhaas, 2014).

Apart from the conventional spectral analysis, the effect of age on WM has often been investigated by the analysis of event-related potential (ERP) or across-trials phase-locking. ERP analysis is based on averaging the EEG profile across multiple trials to obtain a scalp measurable activity (Murray et al., 2008), while across-trials phase-locking focuses on the phase difference between trials. The latter is based on the observation that stimulation can result in a (partial) phase-resetting of the ongoing background EEG, leading to higher phase-locking across trials (Jansen et al., 2003). However, accumulating evidence suggests that the inter-regional synchronization of neuronal activity has an important role in memory formation (Jutras and Buffalo, 2010), and that cognition is a result of interactions among various brain regions that may be spatially separated but functionally linked (Dai et al., 2017). Analysis of inter-regional synchronization of EEG oscillation, which can be considered as indicative of brain functional connectivity, may thus provide additional information to supplement traditional ERP or across-trials analysis.

Furthermore, the association between advanced age and progressive limitations in WM might be due to difficulty in activating the corresponding neural networks (Missonnier et al., 2011). Computational frameworks based on graph theory that model the brain as a complex network have the potential to provide more comprehensive insight into the mechanisms of aging-related cognitive dysfunction. Numerous studies have demonstrated the use of network analysis in identifying and tracking changes in the human brain during normal development and aging, as well as in various neurological and neurodegenerative dementias such as AD (He and Evans, 2010; Sun et al., 2012; Dennis and Thompson, 2014; Sala-Llonch et al., 2015; O'Reilly et al., 2017; Vecchio et al., 2017). Recently, several studies have investigated the topological reorganization of the EEG network in healthy aging (Vecchio et al., 2014; Knyazev et al., 2015; Miraglia et al., 2016); these reports indicate that graph theory can be of use in the analysis of connectivity patterns from EEG, thus facilitating the study of aging-related features of functional connectivity networks in the physiological brain (Vecchio et al., 2014). More interestingly, Dai et al. employed graph theory to characterize the topological properties of brain functional networks during WM tasks, with some meaningful findings (Dai et al., 2017). However, to our best knowledge, network analysis of EEG has never been used to investigate age-related alterations in WM, which are important for uncovering the underlying mechanism of cognitive aging.

Therefore, the aim of the present study was to investigate age-related differences in EEG synchronization and network topology with or without memory load. Two groups of volunteers, one consisting of young students (age: 19–29 years) and the other of older adults (age: 58–70 years), were recruited for a cross-sectional comparison. For each subject, multi-channel EEG was recorded under three conditions, i.e., resting state, 0-back task (control task), and 2-back task (WM task). The EEG signals were filtered into different frequency bands and the phase lag index (PLI) between different EEG channels was used to quantify the inter-regional synchronization. We then explored the topology of the brain functional network constructed with the PLI matrix. Finally, age-related alterations in EEG synchronization and network topology were investigated in different frequency bands and under different conditions.

## Materials and Methods

### Participants

Fifteen undergraduate or graduate students and 13 older adults were recruited. All participants gave their written informed consent before the experiment, and were financially compensated for the experiment regardless of their performance. The experimental protocols were approved by the institutional Ethical Committee of China Pharmaceutical University, and complied with the Declaration of Helsinki.

All participants were healthy right-handed individuals who had normal or corrected-to-normal visual acuity, no history of brain disease, no history of drug, or alcohol abuse, and had not taken any medication in the 2 weeks before the experiment. They declared that they had slept normally the night before the experiment. We performed a baseline assessment of cognitive ability using a Chinese version of the Mini-Mental State Examination (MMSE) (Folstein et al., 1975) for each participant.

All the participants were capable to perform the WM task on an acceptable level. However, data derived from three older adults, who have low quality of EEG recordings due to head moving or frequently eye blinking, were excluded. We thus finally included 15 young (young group; eight females; age: 19–29 years) and 10 older (senior group; five females; age: 58–70 years) participants in this study. The demographics of the included participants are listed in Table 1. Notably, there was a significant difference between the education levels of the two groups and the senior group had a greater standard derivation of education years, compared with the younger participants. No significant difference was found in MMSE scores between the two groups (*t*-test, *p* > 0.05).

**Table 1:** Demographics of the participants.

	**Young**	**Senior**	***P***
*N*	15	10
Age (years)	23.1 ± 2.0	64.0 ± 3.3	0.001
Gender (f/m)	8/7	5/5	n.s.
BMI (kg/m^2^)	20.3 ± 2.1	24.2 ± 3.6	0.002
Education (years)	16.1 ± 1.9	9.3 ± 3.0	0.001
MMSE	29.7 ± 0.9	28.9 ± 3.0	n.s.

*Values are means ± standard derivations, except for Gender. p-values of Student's t-test are listed in the last column, where n.s. represents that there is no significant difference between the two groups*.

### The n-Back WM Task

The classic Sternberg WM task was adopted in the current study. Participants are instructed to watch and respond to a continuous stimuli of symbol “^*^” or Chinese characters (font: Arial, font-size: 58) on a computer screen. Twenty-Five Chinese characters were included according to the following criteria: (1) only nouns; (2) between 4 and 7 strokes; (3) only common words according to the Table of General Standard Chinese Characters, which is issued by the Ministry of Education of the People's Republic of China on 18 June 2013.

**Figure 1:**
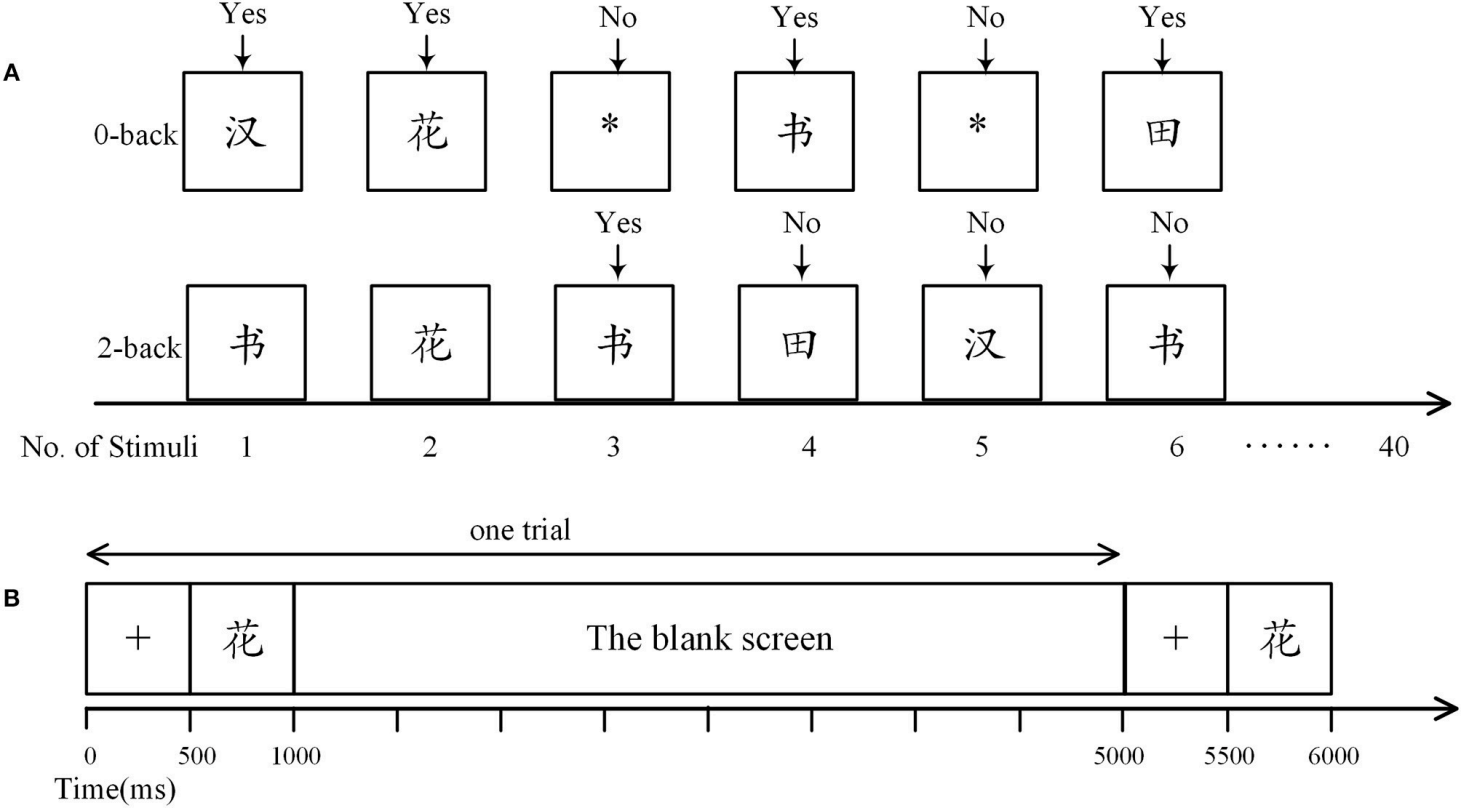
The n-back task. **(A)** Definition of response in the n-back tasks. In the 0-back task, participants were required to press the “Yes” button when a Chinese character arose, otherwise they were to press the “No” button. In the 2-back task, participants were asked to press “Yes” only when the current stimulus matched one of the previous two. Here, the third stimulus was a match as it was identical to the first stimulus. **(B)** Time course of a trial. Each trial began with presentation of a fixation cross (500 ms), followed by a stimulus (500 ms), and then a blank screen (4,000 ms).

Two different cognitive loads were engaged. One is a control task designed based on the 0-back paradigm, in which participants were required to press the “Yes” button when a Chinese Character emerges; and press the “No” button when there is a symbol “^*^” (Figure 1A). The other one is a demanding 2-back task with stimuli constituted of Chinese characters. As shown in Figure 1A, in a 2-back task, the participants were asked to press “Yes” if the current stimulus matches the one 2-time preceded; otherwise press “No.” To eliminate the impact of handedness on the statistical analysis, for half of the participants in both groups, the “Yes” button was defined as the “A” key on a keyboard and “No” button corresponded to the “L” key; while for the others, “Yes” was the “L” key and “No” was the “A” Key. Moreover, participants were suggested to press “A” with their left hands and “L” with their right hands. Note that the other keys on the keyboard were removed.

There were three trial sequences in each n-back task, adding up to 120 trials (including 39 matches). As shown in Figure 1B, in each trial, a 500-ms stimulus was preceded by a 500-ms fixation cross and followed by a 4,000-ms blank screen. Reaction time and accuracy were systematically recorded with E-prime (version 2.0, Psychology Software Tools Inc., Sharpsburg, PA, USA). We did not provide performance feedback. Participants were asked to rest quietly with eyes open for 5 min before the beginning of the n-back task.

### EEG Recording and Preprocessing

EEG signals were recorded using a Brain Vision Recorder (Brain Products Inc., GmbH, Munich, Germany) with 61 surface electrodes placed according to the extended 10–20 system (Nuwer, 1998), a sampling rate of 500 Hz, an online lower cut-off of 0.016 Hz, and an upper cut-off of 70 Hz. Vertical and horizontal electrooculograms were recorded simultaneously from electrodes located above the right eye and the outer canthus of the left eye, respectively. The impedance between each electrode and the skin was kept below 10 *KΩ*. The online reference was the tip of the nose, which was converted to the left and right mastoids (TP9 and TP10) during offline preprocessing. Thus, 59 EEG channels were available for further analysis.

Ocular artifacts were corrected and trials with EEG maximal amplitude exceeding ±60 μV were eliminated using Brain Vision Analyzer (version 2.0, Brain Products Inc.). For EEG signals at rest, an expert examined each continuous 3.5-s segment of artifact-free EEG. For those from the n-back tasks, 3.5-s segments were extracted from each trial beginning with stimulus onset. Then, the theta band (4–8 Hz), alpha band (8–13 Hz), beta band (13–30 Hz), and gamma band (30–45 Hz) were extracted from each segment with finite impulse response filters provided by MATLAB (Mathworks Inc., Natick, MA, USA). This filter processes EEG signals forwards and backwards, yielding EEG waves with zero phase distortion. After discarding both the first and the last 0.5 s of each wave, we prepared 2.5-s segments in the theta, alpha, beta, and gamma bands for the following analysis.

### Network Computation and Metrics

For each participant, after preprocessing and regardless of the conditions, the network construction and computation were performed on each 2.5-s EEG segment in the theta, alpha, beta, and gamma bands. When using graph theory to construct a network of brain functional connectivity, the brain regions are commonly considered as network nodes and the functional connections as edges (Rubinov and Sporns, 2010). In the present study, for each 2.5-s multi-channel EEG segment, we took all the EEG channels as the network nodes, and then used the PLI between nodes to construct the connectivity matrix. As the choice of the threshold may have a strong effect on the estimation of the network metrics (Bullmore and Bassett, 2011), a fully connected, weighted, and undirected network (WUN) was built directly from the PLI matrix. Recently, such a WUN framework was used in various studies (Xue et al., 2014; Vecchio et al., 2015, 2016; Gong et al., 2017). The networks were then analyzed in terms of their node-to-node connectivity and their local and global network characteristics. The network metrics were obtained with the MATLAB Brain Connectivity Toolbox (Rubinov and Sporns, 2010; Whitfield-Gabrieli and Nieto-Castanon, 2012), which is widely used in graph theoretical analysis of the brain (Hong et al., 2016; McKenna et al., 2016; Soman et al., 2016).

#### Inter-Regional Functional Connectivity

PLI was used as a measure of inter-regional functional connectivity in this study. The major aim of using PLI is to obtain reliable estimates of phase synchronization that are invariant against the presence of common sources, such as volume conduction and/or active reference electrodes in the case of EEG (Stam et al., 2007; Hardmeier et al., 2014).

In short, PLI is an index of the asymmetry in the distribution of phase differences calculated from the instantaneous phases of two time series. For a real-valued signal *s*(*t*), one can define its Hilbert transform and analytic signal as shown in Equations (1, 2), respectively.

(1) $$\begin{array}{r} \hat{s}\left(t\right)=\int_{-\infty }^{+\infty }s\left(\tau \right)h\left(t-\tau \right)d\tau =\frac{1}{\pi }\int_{-\infty }^{+\infty }\frac{s\left(\tau \right)}{t-\tau }d\tau \end{array}$$

(2) $$\begin{array}{r} z\left(t\right)=s\left(t\right)+i\times \hat{s}\left(t\right)=A\left(t\right)e^{i\emptyset \left(t\right)} \end{array}$$

Here, *A*(*t*) and ∅(*t*) are the instantaneous amplitude and phase (IP) of *s*(*t*), respectively. Considering two simultaneously recorded time series *p*(*t*) and *q*(*t*), their IP difference Δφ_*pq*_(*t*) can be defined as in Equation (3).

(3) $$\begin{array}{r} \Delta \varphi_{pq}\left(t\right)=\emptyset_{p}\left(t\right)-\;\emptyset_{q}\left(t\right) \end{array}$$

Then, the PLI of the two time series *p*(*t*) and *q*(*t*) can be obtained as shown in Equation (4):

(4) $$\begin{array}{r} \text{PLI}=|\;<\text{sign}\left[\text{sin}\left({\Delta \varphi }_{pq}\left(\text{t}\right)\right)\right]>| \end{array}$$

where sign and sin stand for signum and sinusoidal function, respectively, and < > and | | denote the mean and the absolute value, respectively. The value of PLI ranges between 0 and 1, with 0 indicating a total absence of synchronization, and its maximal value of 1 corresponds to a perfect non-zero phase locking (Stam et al., 2007; Hardmeier et al., 2014). By taking each EEG-channel signal as a real-valued time series, node-to-node PLI values were calculated and formed a 59 × 59 connectivity matrix. Additionally, the average PLI over all the node pairs was computed and used as a global EEG synchronization measure.

#### Clustering Coefficient

The clustering coefficient, a classic metric in graph theory, is an index of local structure. Locally, the *C*_i_ of a node *i* corresponds to the fraction of triangles around itself in a binary graph. This concept was generalized to weighted complex networks by Onnela et al. (2004). In the present study, it was calculated using Equation (5).

(5) $$\begin{array}{r} C_{\text{i}}=\frac{\sum_{j=1,h=1}^{n}{\left({\text{PLI}}_{ij}{\text{PLI}}_{ih}{\text{PLI}}_{jh}\right)}^{\frac{1}{3}}}{\sum_{j=1,j\neq i}^{n}{\text{PLI}}_{ij}\left(\sum_{j=1,j\neq i}^{n}{\text{PLI}}_{ij}-1\right)} \end{array}$$

Here, *n* represents the number of nodes in the analyzed WUN. Hence, the average clustering coefficient for the network (denoted as *C* in the following) reflects, on average, the prevalence of clustered connectivity around individual nodes. It is generally used an indicator of the functional segregation in the brain (Rubinov and Sporns, 2010; Whitfield-Gabrieli and Nieto-Castanon, 2012).

#### Characteristic Path Length

The characteristic path length, defined as the mean of the geodesic lengths over all couples of nodes, is another fundamental metric of networks. It is also the most commonly used measure of functional integration (Rubinov and Sporns, 2010). In the present study, it was denoted as *L* and calculated according to Equation (6):

(6) $$\text{L}=\frac{1}{n(n-1)}{\sum^{\text{​}}}_{i=1}^{n}{\sum^{\text{​}}}_{j=1,\;j\neq i}^{n}{\sum^{\text{​}}}_{PLI_{uv}\in g(i↔j)}f(PLI_{uv})$$

where *n* is the number of nodes in the analyzed WUN, *f* is a map (here, an inverse function) from weight to length, and **g**(**i ↔ j**) represents the shortest weighted path between nodes *i* and *j*.

#### Small-World Coefficient

Functional segregation in the brain relates to the ability to perform specialized processing, while functional integration is the ability to rapidly combine specialized information from distributed brain regions. There is a plausible hypothesis that an optimal balance between functional segregation and integration should lead to more efficient processing of information (Sporns and Honey, 2006). Therefore, a measure called the small-world coefficient (SW) has been proposed to quantify this balance by comparing the normalized clustering coefficient and characteristic path length of a network. First, a large set of randomized networks should be generated, based on the original network. The next step is to calculate the average clustering coefficient and characteristic path length of the randomized networks, denoted *C*_rand_ and *L*_rand_, respectively. Finally, the SW can be obtained as in Equation (7):

(7) $$\begin{array}{r} \text{SW}=\frac{C/C_{\text{rand}}}{L/L_{\text{rand}}} \end{array}$$

where *C* and *L* are the clustering coefficient and characteristic path length, respectively, of the original network.

### Statistical Analysis

Age-related and task-evoked changes in the inter-regional functional connectivity, as well as the global and local network metrics described above, were analyzed using MATLAB. For each frequency band, all network metrics were obtained by averaging the results obtained from all segments for a given condition. The statistical analysis was performed on those average values.

Non-parametric methods were used for statistical evaluations because the data did not meet the assumptions for normality or homogeneity. In order to investigate the aging effect, an analysis of partial correlation was conducted between EEG measures and group (young or senior) by controlling educational level, as there were substantial differences in years of education. Meanwhile, task-evoked differences in EEG measures among the three conditions were assessed using Friedman's non-parametric test; if the difference was significant (*p* < 0.05), *post-hoc* analyses were performed using pair-sample Wilcoxon sign rank (WSR) tests with Tukey-Kramer adjustment.

Moreover, a Mann-Whitney non-parametric U (MWU) test was used to assess the age-related difference of behavioral performance (response accuracy and reaction time) in each n-back task. For each age group, paired-sample WSR tests were used to check whether there was any difference in behavioral performance when different n-back tasks were engaged. Furthermore, the association between the network metrics and the behavioral performance in the WM task, was examined by a partial correlation analysis, controlling age and education.

The false discovery rate (FDR) was controlled at a significance level of 0.05 (Benjamini and Hochberg, 1995) in cases involving multiple comparisons, i.e., in the analyses of node-to-node PLIs and local clustering coefficients.

## Results

### Behavioral Results

The senior group exhibited significantly lower response accuracy and longer reaction time compared with the younger group (MWU test, *p* < 0.05) on both the 0-back and 2-back tasks, indicating worsening behavioral performance in cognitive tasks with aging (Table 2). Moreover, although both groups showed significantly increased reaction time during the 2-back task (in which WM was engaged) compared with the 0-back task, there were no significant differences in response accuracy for either group (paired-sample WSR tests, *p* > 0.05).

**Table 2:** Response accuracy (%) and reaction time (mean ± SD) for both groups on the 0-back and 2-back tasks.

	**Response accuracy (%)**			**Reaction time (ms)**		
	**Young**	**Senior**	**MWU**	**Young**	**Senior**	**MWU**
0-back	0.95 ± 0.03	0.88 ± 0.10	0.03	497 ± 109	660 ± 127	<0.01
2-back	0.96 ± 0.03	0.73 ± 0.20	<0.01	743 ± 244^**^	1092 ± 527^**^	0.03

** *p < 0.01 for the paired-sample WSR test between the performances of 2-back task and the control task. MWU represent for the Mann-Whitney non-parametric U test to assess the age-related difference of behavioral performance in each n-back task*.

### Inter-Regional Functional Connectivity

**Figure 2:**
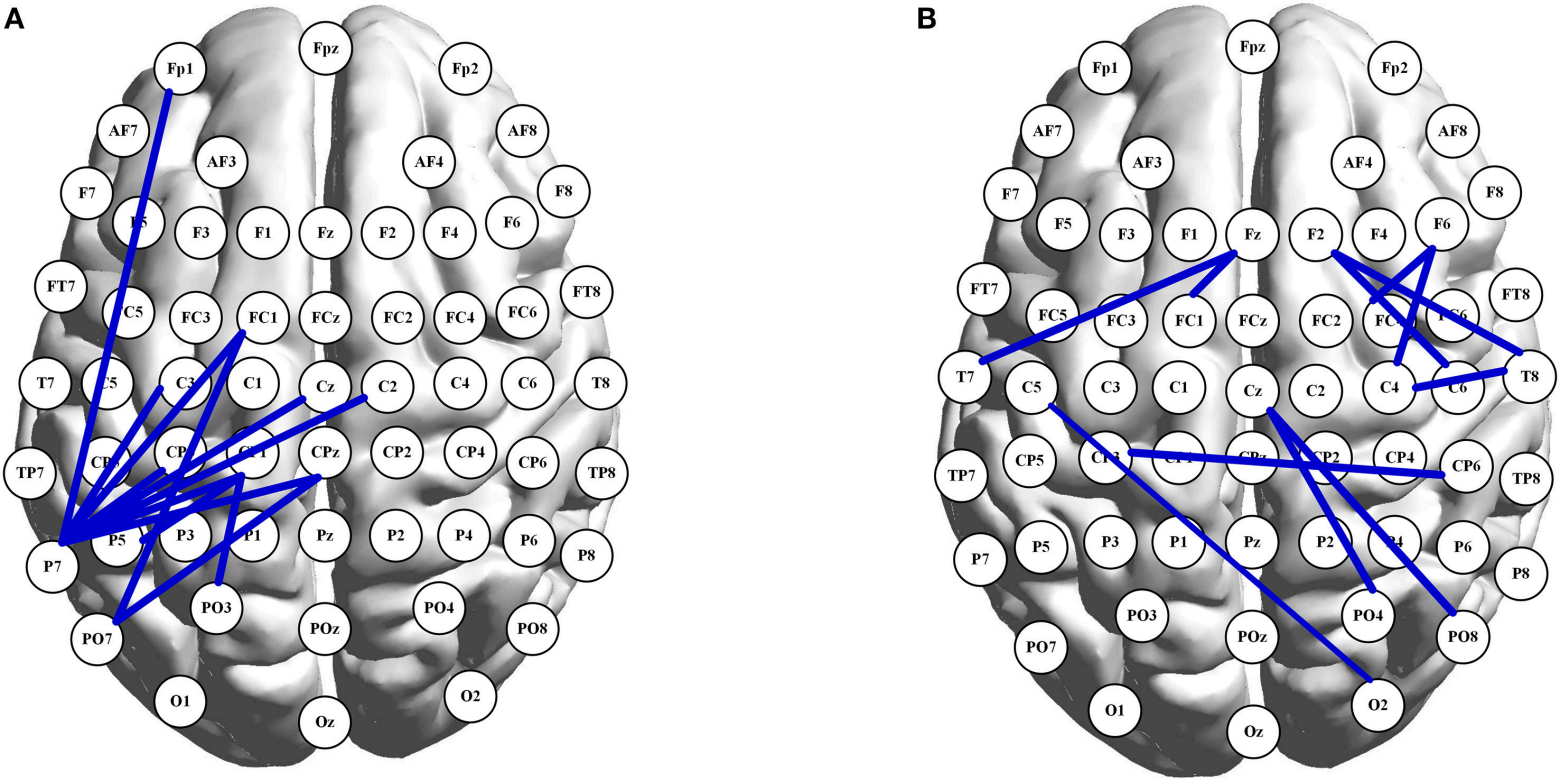
Graph presentation of node-to-node connections in the alpha band. In **(A)**, each shown connection has a significant decline in PLI with aging under 2-back condition. In **(B)**, illustrated connections have significant larger values of PLI under 0-back condition than those under 2-back task, for the young adults. The brain map was drawn using the BrainNet Viewer toolbox (version 1.61, Beijing Normal University, China) (Xia et al., 2013).

PLI matrices of 59 × 59 nodes were used to study the age-related and task-evoked changes in inter-regional connectivity. No significant alterations in node-to-node connectivity were found when controlling the FDR. Nevertheless, when the significance level was set to 0.001 without any correction, in the alpha band, 12 pairs of nodes showed significant age-related changes (all decreasing with aging) in connectivity under the 2-back condition. Furthermore, 11 pairs exhibited significant task-evoked alterations in both n-back tasks (all decreasing with the memory load), but only for the younger adults. Those edges are illustrated in the graph representation of the brain shown in Figure 2. As can be seen in Figure 2A, for the 2-back task, age-related decreases in alpha-band functional connections were mainly concentrated in the left hemisphere, starting and ending in the left parietal nodes (in particular, the P7 node). In other frequency bands, no more than five pairs of nodes retained their connectivity, regardless of whether age-related or task-evoked changes were being investigated, when the significance level was set to 0.001 without any correction.

However, a significant correlation (*p* = 0.003, partial correlation, controlling education) between age group and average PLI was observed in the theta band during the 2-back task, but not for any other frequencies or conditions. That is, the senior group had a higher average PLI (0.223 ± 0.012) compared with the young group (0.205 ± 0.005), indicating an age-related increase in inter-regional synchronization of EEG theta activity in WM. Moreover, for the young participants, we found a significant task-related effect on the average PLI of EEG beta activity (*p* = 0.03, Friedman's non-parametric test). According to *post-hoc* analysis, the young participants showed enhanced global synchronization of EEG beta activity when performing the 2-back task (0.117 ± 0.002), compared with at rest (0.109 ± 0.003). Note that PLI values are presented here as mean ± standard error.

### Age-Related Differences in Network Metrics

**Figure 3:**
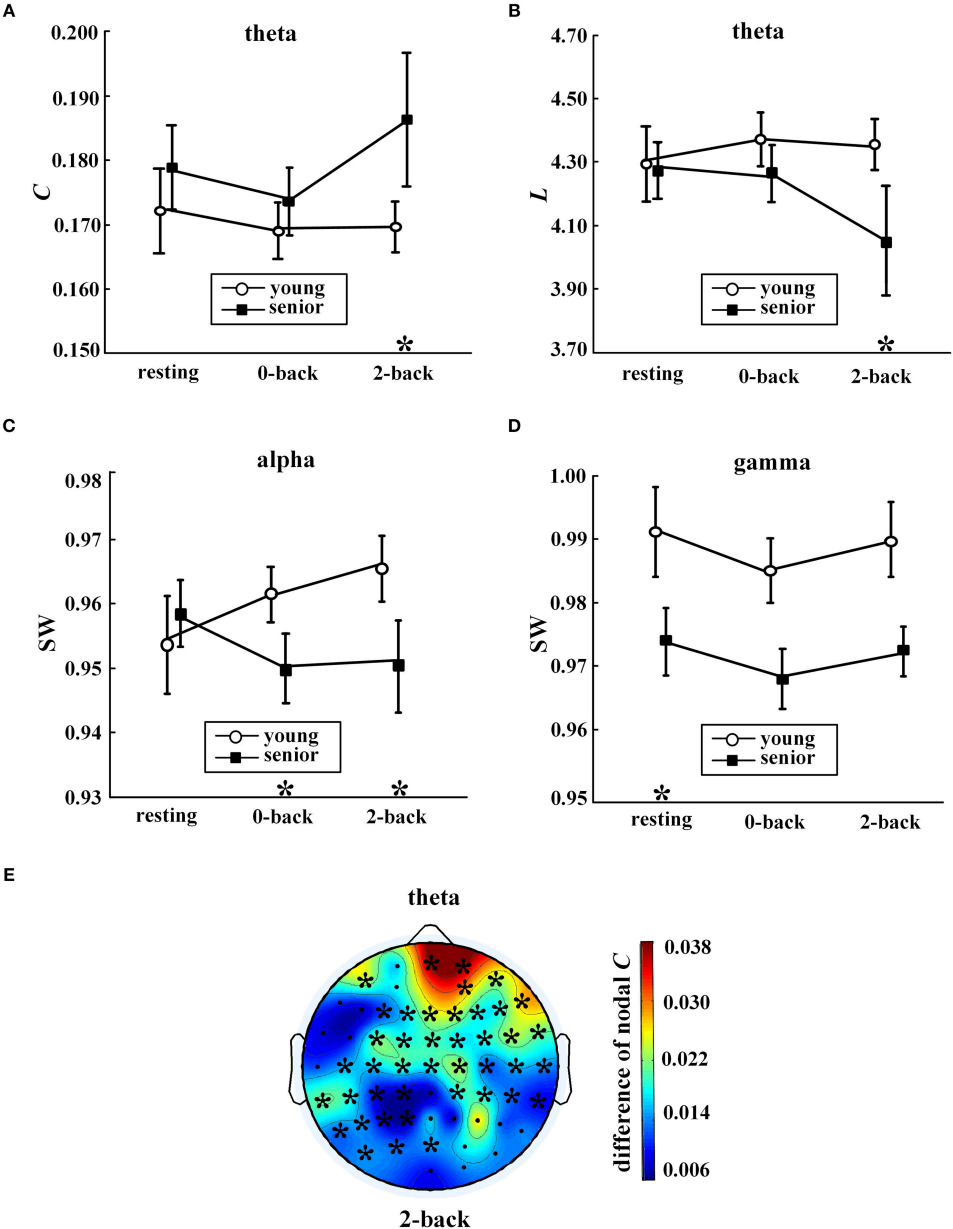
Age-related differences in network metrics. Values (mean ± standard error) are shown for **(A)** average clustering coefficient in the theta band, **(B)** characteristic path length in the theta band, **(C)** small-world coefficient in the alpha band, and **(D)** small-world coefficient in the gamma band. The symbol “^*^” above a condition represents a significant correlation between age group and metrics (*p* < 0.05, partial correlation) during this condition. In **(E)**, the distribution of differences in nodal clustering coefficients in the theta network between the senior and young group (senior minus young) during the 2-back condition is shown. The symbol “^*^” indicates a significant correlation between age group and nodal C (partial correlation analysis with FDR controlled). The EEG map was drawn using the Topoplot toolbox in MATLAB.

During the 2-back task, age group was significantly correlated with *C* of the theta network (*p* = 0.002), *L* of the theta network (*p* = 0.003), and SW of the alpha network (*p* = 0.009). As shown in Figures 3A–C, the main alterations in network topology observed in WM with aging were a prominent increase in *C* and decrease in *L* of the theta network, and a significant decrease in SW of the alpha band.

Moreover, as shown in Figures 3C–D, compared with the young group, the senior group exhibited significant declines in SW in the alpha band during the 0-back task (*p* = 0.01) and in the gamma band during resting state (*p* = 0.006).

Furthermore, analysis was conducted to investigate the correlation between the age group and nodal *C*, as shown in Figure 3E. A significant aging effect (*p* < 0.05, partial correlation and FDR corrected) was found only in the theta network during the 2-back task. When WM was engaged, widespread aging-related increases in nodal *C* in the theta network were found across multiple locations, in particular, in the prefrontal regions.

### Task-Evoked Alteration in Network Metrics

**Figure 4:**
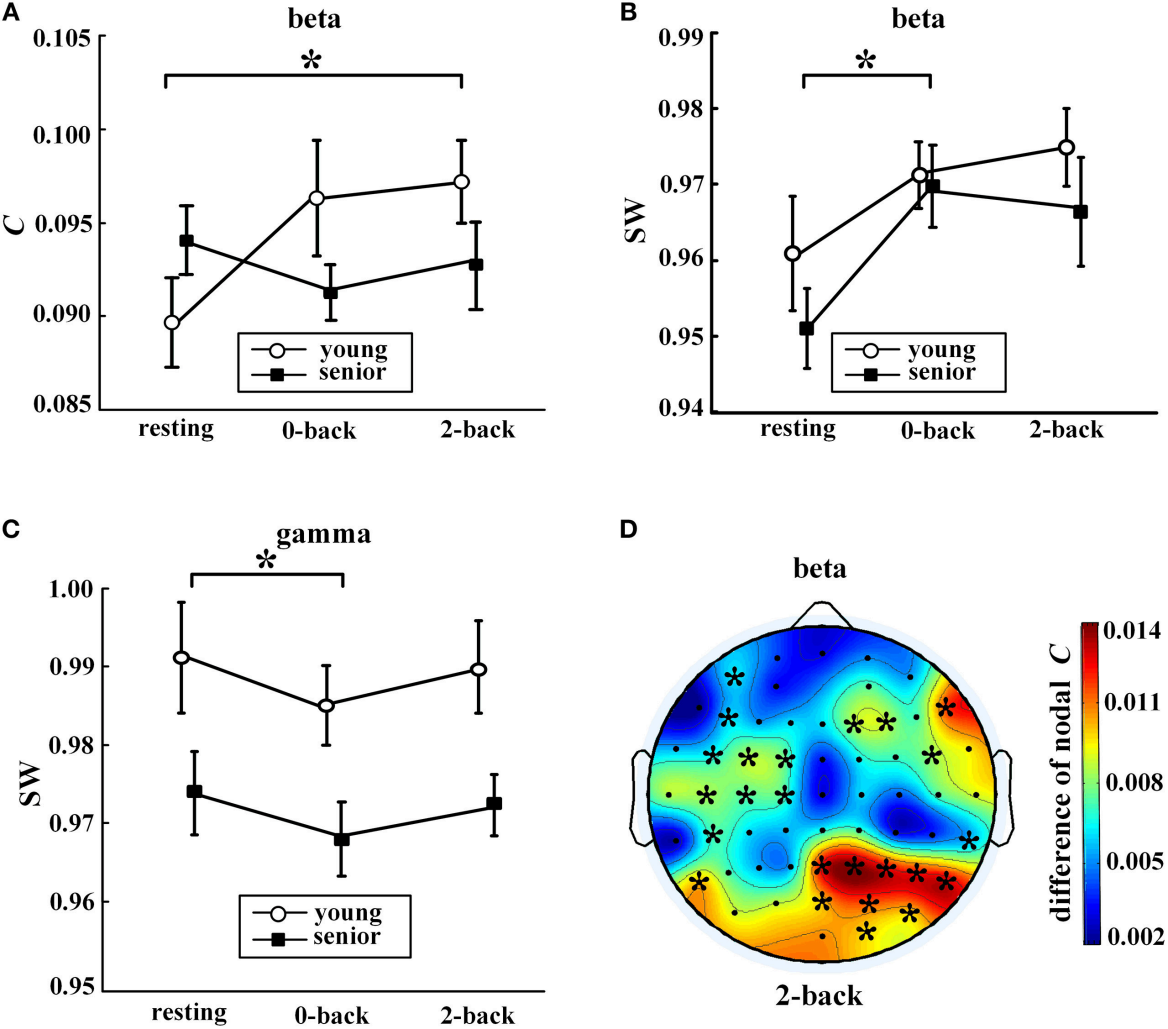
Task-evoked alterations in network metrics. In **(A–C)**, values (mean ± standard error) indicate significant task-evoked alterations in **(A)** average clustering coefficient in the beta band, **(B)** small-world coefficient in the beta band, and **(C)** small-world coefficient in the gamma band. The symbol “^*^” above a horizontal line corresponds to a between-condition difference for a certain group (*p* < 0.05, Friedman's non-parametric test). Part **(D)** shows the distribution of the differences in nodal clustering coefficients of the beta network between the 2-back task and resting state for the young group (2-back minus resting). The symbol “^*^” indicates a significance difference (WSR test) that remained after FDR controlling. The EEG map was drawn using the Topoplot toolbox in MATLAB.

Task-evoked alterations in network metrics were observed in the fast EEG bands, i.e., the beta band and the gamma band, as shown in Figure 4. Compared with the resting state, the older subjects exhibited a significant increase in SW of the beta network and a decline in SW of the gamma network during the 0-back task. For the younger subjects, the only significant increase was in *C* of the beta network during the 2-back task.

Moreover, as shown in Figure 4D, task-induced alteration of nodal *C* was observed only in the beta band and only for the younger adults. During the 2-back task, the younger participants exhibited significantly increased nodal *C* compared with the resting state in many regions, especially in the right parietal-occipital areas.

### Correlation Between WM Performance and EEG Measures

We investigated the associations between EEG metrics and behavioral performance during a WM task, i.e., the 2-back task in the current study, across all participants while controlling age and education. As shown in Table 3, response accuracy was positively correlated with the average PLI of EEG gamma activity and *C* of the gamma network, and negatively correlated with SW of the theta network and SW of the gamma network. The average PLI and *C* in the theta band showed positive correlation with reaction time, whereas *L* in the theta band exhibited a negative correlation.

**Table 3:** Correlation coefficients between EEG measures and behavioral performance during 2-back task.

	**Response accuracy (%)**				**Reaction time (ms)**			
	**Theta**	**Alpha**	**Beta**	**Gamma**	**Theta**	**Alpha**	**Beta**	**Gamma**
Average PLI	−0.114	0.062	0.006	0.438^*^	0.522^*^	0.261	−0.129	0.235
*L*	0.096	0.054	0.033	−0.387	−0.494^*^	−0.371	0.226	−0.225
*C*	−0.103	0.061	−0.010	0.426^*^	0.501^*^	0.261	−0.107	0.239
*SW*	−0.512^*^	−0.265	−0.270	−0.416^*^	0.395	0.051	0.017	−0.107

* *p < 0.05*.

**Figure 5:**
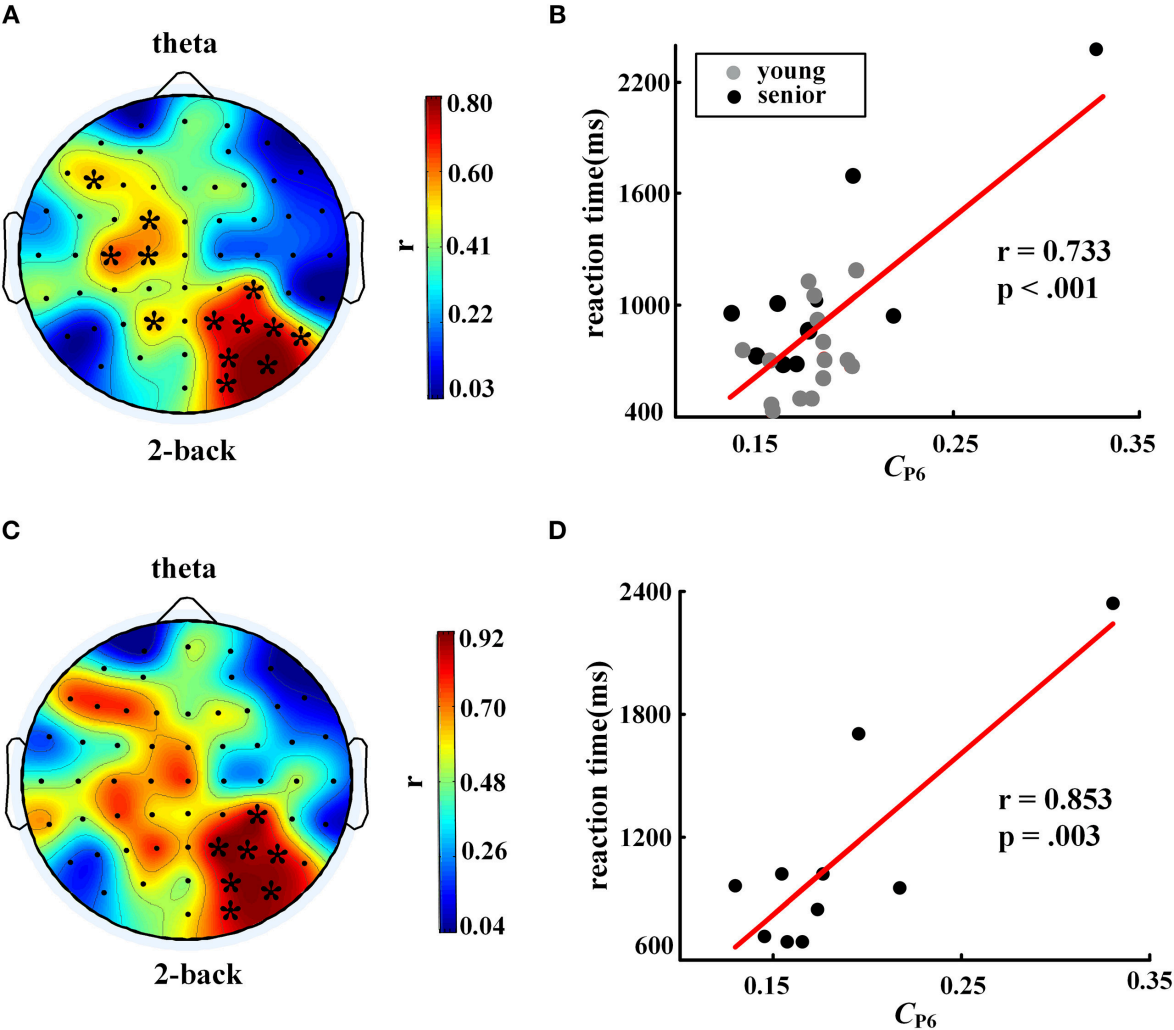
Correlation between nodal clustering coefficient and reaction time in 2-back task. The left column shows distributions of the correlation coefficients, and the right column shows scatter plots of the correlations in electrode P6. The top row, **(A)** and **(B)**, shows results for all participants (controlling age and education), while the bottom row, **(C)** and **(D)**, shows results for the senior group only (controlling education). The symbol “^*^” represents a significant correlation after FDR correction. The EEG map was drawn using the Topoplot toolbox in MATLAB.

When the nodal *C* was considered, we found a significant positive correlation between reaction time in the 2-back task and nodal *C* (partial correlation analysis with FDR corrected, controlling age and education years), as shown in Figures 5A,B, especially in the right posterior parietal lobe. This phenomenon became more prominent when only the older subjects were included in the correlation analysis. As shown in Figures 5C,D, for the older individuals, a larger *C* in the P6 node was corresponding to a longer time consumed in the WM task.

## Discussion

In this study, we analyzed age-related and task-evoked variations in EEG inter-regional synchronization and network topology. Typical cross-sectional comparisons between young and old adults were designed, and multi-channel EEGs were recorded and analyzed for subjects at rest and while performing n-back tasks. The key findings were as follows. (1) Age-related alterations were more prominent during the 2-back task, especially in the theta band. (2) For the young group, task-induced changes were observed in the beta band between the 2-back and resting conditions; while for the senior group, the 0-back task evoked significant alterations in network topology in both the beta and gamma band. (3) During the 2-back task, the EEG metrics in the theta and gamma bands showed significant correlations with behavioral performance. Overall, our results suggest that different EEG frequencies have different functional correlates in cognitive aging, as discussed below.

### Theta Oscillations Reflect a Compensatory Mechanism in Cognitive Aging

WM comprises a large number of cognitive processes, with different neural correlates involving different brain areas, which need to be coordinated to monitor and control complex WM tasks. Theta activity is generally considered to be responsible for the control of WM functions and has been proposed as an underlying mechanism for this integration in WM (Sauseng et al., 2010).

In the current study, compared with the younger adults, the senior group exhibited stronger inter-regional synchronization of EEG theta activities. The theta-band functional connectivity network became more globally integrated and also showed widespread enhanced ability for specialized processing, especially in the prefrontal area. Moreover, the EEG metrics in the theta band showed significant correlations with behavioral performance in the 2-back task, when WM was engaged. For the older individuals, the nodal clustering coefficient of the theta network in the right parietal area was positively correlated with reaction time in the 2-back task, consistent with previous reports (Sala-Llonch et al., 2014; Dai et al., 2017). These findings support the integrative role of the theta band in WM tasks, which could be attributed to compensatory activation, a characteristic of normal aging (Phillips and Andres, 2010).

Evidence from positron emission tomography and fMRI studies have shown that, as well as task-related “under-activation” in older adults relative to younger adults in some regions (e.g., the hippocampus), “over-activation” can be observed in many other regions, particularly the frontal-parietal regions, whose involvement in WM is well-documented (Cappell et al., 2010; Kennedy et al., 2015). According to the compensation-related utilization of neural circuits hypothesis (Reuter-Lorenz and Cappell, 2008), over-activation might occur because more neural resources are engaged by aging brains to accomplish computational goals that would be completed with fewer resources by younger brains (Cappell et al., 2010). In the present study, compared with the 0-back task, in which sustained attention but no WM engagement is required, both groups had longer response times in the 2-back task. However, response accuracy was stable in the younger adults, whereas it had a decreasing trend in the senior group (*p* = 0.07, paired-sample WSR test). Accordingly, we speculated that the memory load in the 2-back task might be within the capacity of WM for young adults but beyond that of the elders; the latter are more likely to require the allocation of additional neural resources, leading to a widespread increase in the nodal clustering coefficient, and improved synchronization of inter-regional EEG theta activity and functional integration of the theta network. Meanwhile, the older individuals, especially those with longer reaction times in the 2-back task, might have made greater efforts than the young to utilize the engaged resources in the frontal and parietal-occipital areas.

### Role of Alpha Activity in Attentional Control

A central role has been proposed for alpha activity as an attentional suppression mechanism, including in WM (Klimesch, 2012; Roux and Uhlhaas, 2014). That is, alpha oscillation underlies the suppression of spurious brain activities and the inhibition of irrelevant information in cognitive tasks (Dai et al., 2017; Guevara et al., 2018). As the desynchronization of this band is considered to be associated with enhanced attention (Sauseng et al., 2005; Guevara et al., 2018), the results shown in Figure 3A suggest that, with normal aging, the left parietal region might participate more in attentional control in WM.

Furthermore, in the present study, significant age-related topological reorganizations of the EEG alpha-band network were found during both n-back task conditions, but not in the resting state, which might reflect the role of alpha activity in attention control. Compared with the young group, the senior group exhibited a decline in the small-world coefficient, which has been proposed as a measure for the balance between functional integration and segregation (Sporns and Honey, 2006). This suggests a possible association between degraded network architecture in the alpha-band network and aging-related changes in attention. However, further confirmation of this association is required, as the small-worldness measure was seldom applied to fully connected and weighted networks. Small-world networks usually have a small-world coefficient far >1 (Rubinov and Sporns, 2010), while in the current study, as well as in similar studies (Miraglia et al., 2016, 2017; Vecchio et al., 2016), the obtained small-world coefficients were very close to 1. In this study, we thus illustrated the results relating to the small-world coefficient with limited discussion.

### Absence of Task-Evoked Beta Response in Aging

The beta band has been shown to have a role in inter-neuronal communication of inhibitory networks and high executive demands (Guevara et al., 2018). Synchronization in the beta band has been correlated with sensory processing (Singer, 1993) and increased attentiveness (Makeig and Jung, 1996). Sarnthein et al. made the interesting observation that beta synchronization (19–32 Hz) increased during both perception and retention intervals in WM tasks (Sarnthein et al., 1998).

In the present study, no significant age-related changes were found in the beta band, whereas task-evoked alterations were prominent in this band. For the younger adults, with respect to the resting state, increases in the local density of connections were observed in many regions, indicating a significantly greater potential for functional segregation in the WM network. However, this response seemed to be absent in WM in the senior group. Our results suggest that the inhibition-related processes in WM are more demanding and require greater specialization in the beta band functional connectivity network, and that the aging brain might fail to respond to this requirement.

### Correlation Between Gamma Oscillation and Memory Performance

Synchronization of gamma oscillations is thought to be generically involved in the maintenance of WM information (Roux and Uhlhaas, 2014; Lundqvist et al., 2018). The greater synchronization of the fast bands, especially within the gamma range, could indicate a greater active maintenance of information that is necessary for the evocation of perceived stimuli (Guevara et al., 2018). This seems to be a plausible interpretation of our observation that enhanced synchronous activity in the gamma band was correlated with increased response accuracy in the 2-back task.

One of most interesting findings of this study was the observation that the significant correlation between behavioral performance and EEG metrics existed only in the theta and gamma bands. This might support the idea of gamma oscillations being nested in theta cycles during WM. According to this model, individual items to be held in WM are represented by single gamma periods, and these gamma cycles are nested into a theta period. The phase relation between gamma and theta oscillations can thus code the sequence of items. Such modulation in rhythmic synchronization in the gamma- and theta-bands has shown to be related to memory performance, and interesting relationships have been described between these oscillations, suggesting a mechanism for inter-areal coupling (Jutras and Buffalo, 2010). However, further work is required to determine why the opposite relationship between memory performance and EEG metrics, except for the small-world coefficient, was observed in these two frequency bands.

### Limitations

In this study, frequency band-dependent alterations in EEG synchronization and network topology during cognitive aging were investigated. This study had some limitations. First, the sample size was small and the two groups used for the cross-sectional comparison had imbalanced educational levels. Although, we included years of education as a covariate in the statistical analysis, the results might have been influenced by the small sample size together with the relatively large number of variables in the statistical models. Another limitation relates to the insufficient evaluation of cognition, which was based only on MMSE without imaging to check for potential brain atrophy. Use of structural MRI is suggested for future work. In spite of these limitations, our findings contribute to the understanding of age-associated changes in memory and the role of EEG oscillations in WM. Future studies are encouraged to incorporate with larger samples and further types of work (for example, 1-back and 3-back tasks). Moreover, this proposed approach is expected to work/associate together with conventional spectral, ERP and across-trials analysis, and to provide in-depth investigation for the cognitive aging.

## Author Contributions

FH, XX, C-KP, JZ, and CW designed research; FH and CL performed research; CL and ZY analyzed data; FH, CL, ZY, and AY wrote the article. FH and CL contributed equally to this work.

### Conflict of Interest Statement

The authors declare that the research was conducted in the absence of any commercial or financial relationships that could be construed as a potential conflict of interest.
